# A new method for estimating patient body weight using CT dose modulation data

**DOI:** 10.1186/s41747-017-0028-z

**Published:** 2017-12-04

**Authors:** Dominic Gascho, Lucia Ganzoni, Philippe Kolly, Niklaus Zoelch, Gary M. Hatch, Michael J. Thali, Thomas D. Ruder

**Affiliations:** 1 Department of Forensic Medicine and Imaging, Institute of Forensic Medicine, University of Zurich, 8057 Zurich, Switzerland; 2 Department of Clinical Research, University of Bern, 3008 Bern, Switzerland; 3 Hospital of Psychiatry, Department of Psychiatry, Psychotherapy and Psychosomatics, University of Zurich, 8032 Zurich, Switzerland; 4 Center for Forensic Imaging, Departments of Radiology and Pathology, University of New Mexico School of Medicine, Albuquerque, NM 87102 USA; 5 Institute of Diagnostic, Interventional, and Pediatric Radiology, University Hospital Bern, 3010 Bern, Switzerland

**Keywords:** Body weight, Computed tomography, Dose modulation, Emergency radiology, Virtopsy

## Abstract

**Background:**

Body weight (BW) is a relevant metric in emergency care. However, visual/physical methods to estimate BW are unreliable. We have developed a method for estimating BW based on effective mAs (mAs_eff_) from computed tomography (CT) dose modulation.

**Methods:**

The mAs_eff_ of CT examinations was correlated with the BW of 329 decedents. Linear regression analysis was used to calculate an equation for BW estimation based on the results of decedents with a postmortem interval (PMI) < 4 days (n = 240). The equation was applied to a validation group of 125 decedents. Pearson correlation and *t*-test statistics were used.

**Results:**

We found an overall strong correlation between mAs_eff_ and BW (*r* = 0.931); *r* values ranged from 0.854 for decedents with PMI ≥ 4 days to 0.966 for those with PMI < 4 days; among the latter group, *r* was 0.974 for females and 0.960 for males and 0.969 in the presence and 0.966 in the absence of metallic implants (all correlations with *p* values < 0.001). The estimated BW was equal to 3.732 + (0.422 × mAs_eff_) – (3.108 × sex index), where the sex index is 0 for males and 1 for females. The validation group showed a strong correlation (*r* = 0.969) between measured BW and the predicted BW, without significant differences overall (*p* = 0.119) as well as in female (*p* = 0.394) and in male decedents (*p* = 0.196). No outliers were observed.

**Conclusions:**

CT dose modulation is a rapid and reliable method for BW estimation with potential use in clinical practice, in particular in emergency settings.

## Key points

- CT using dose modulation can be used to estimate BW
- Effective mAs values showed strong correlation with measured BW
- An equation can be calculated to estimate BW
- This method has potential use in emergency settings

## Background

The estimation of body weight (BW) is a relevant issue in emergency care as accurate drug dosing [1, 2], such as in thrombolysis of acute ischaemic stroke [3, 4] or the dosage of contrast media [5, 6], is related to BW. Patients in emergency care may be unresponsive and thus unable to state their BW, and visual estimates of BW are unreliable [1, 7, 8]. A few methods to estimate BW (beyond a simple visual estimate), applicable to both the living and the dead, are mentioned in the literature [2, 9]. Recording BW of a decedent prior to autopsy is a standard procedure in forensic medicine [10, 11]. However, these methods yield moderate accuracy [2] or are at least technically challenging and time consuming [9]. Therefore, developing a new approach for BW estimation is a relevant issue.

At our institute of forensic medicine, we use a calibrated floor scale to measure BW accurately. Additionally, each decedent undergoes computed tomography (CT) as a supplement to autopsy. Postmortem CT exams utilize tube current modulation [12]. A main purpose of tube current modulation is the adjustment of dose exposure to body anatomy, yielding almost constant image noise along the scan [13, 14]. By measuring beam attenuation during the localizer scan, automated dose modulation calculates a dose distribution based on a reference value of mAs, i.e. a user-selected reference mAs value (mAs_ref_), and on body anatomy. The shape and size of a typical adult person with a BW of 70–80 kg served as reference for this technique. Thus, increased tube current is applied for overweight people (higher attenuation detected in the localizer) and decreased tube current for underweight people (lower attenuation detected in the localizer) [15]. Since dose modulation adjusts dose exposure according to individual deviations from the ideal patient and the reference standard of 70–80 kg [13, 14], we assumed that adjusted mAs values over the whole body (effective mAs, mAs_eff_) may correlate with BW of adults.

The aim of this study was to evaluate the correlation between mAs_eff_ values and measured BW to develop a linear regression equation for BW estimation in adults.

## Methods

### Study population

Scan data were acquired as part of a forensic judicial investigation. Data usage is conformant with Swiss laws and ethical standards as approved by the Ethics Committee of the Canton of Zurich (written approval, KEK ZH-Nr. 2015-0686).

We reviewed all cases that underwent postmortem whole body CT between September 2015 and June 2016 (n = 459). Exclusion criteria were: decedents with an age < 17 years (n = 15), use of non-standard scan parameters in the context of research purposes (n = 20), and dismembered corpses (n = 95). Thus, the final study population consisted of 329 decedents (105 females and 224 males) with a mean age of 59.0 years (standard deviation [SD] 59.0 ± 18.0 years; range 18–95 years). Taking into consideration that decomposition- or putrefaction-related changes usually start to appear after 72 h after demise [16], the study population was divided into two groups with different postmortem interval (PMI): 240 decedents with a PMI < 4 days (78 females and 162 males) and 89 decedents with a PMI ≥ 4 days (27 females and 62 males). The former group was further subdivided into subgroups according to gender (78 female and 162 males) as well as according to the presence of metallic medical implants (38 with and 202 without). After evaluation of the data distribution using the Kolmogorov–Smirnov test, Pearson’s correlation coefficient (*r*) was used to assess the correlation between measured BW and mAs_eff_ for each group and subgroup. The *p* values of the correlations were also calculated.

Linear regression analyses were used to create a model to be used to estimate BW based on mAs_eff_, taking into consideration sex and implants. The group with PMI < 4 days was used for the calculation of an equation for BW estimation; therefore, the calculated constant and the unstandardized coefficients (B) were used to develop the equation. According to the multivariate linear regression analysis, sex and/or implants were taken into account for the equation. Further, the standard error of the estimates (SEE) was calculated. The final equation was applied on a validation group, which included all cases between December 2016 and March 2017 (n = 204). Exclusion criteria were the same as mentioned above with the addition of a PMI ≥ 4 days. The final validation group consisted of 125 decedents (43 females and 82 males) with a mean age of 56.4 years (SD 56.4 ± 18.3 years; range 18–96 years). After evaluation of data distribution using the Kolmogorov–Smirnov test, the Student *t*-test was applied to reveal significant differences between actual BW and BW predicted by the linear regression equation.

All CT exams utilized automated dose modulation and were performed at the request of local legal authorities.

### Imaging protocol

Postmortem CT was performed on a 128-slice scanner (SOMATOM Definition Flash, Siemens Healthcare, Forchheim, Germany) using the dose modulation technique (CARE Dose 4D™, Siemens Healthcare, Forchheim, Germany). The CT scan protocol included frontal and lateral localizer topogram or scout view using 120 kVp and 35 mA. Dose modulation was based on attenuation measurements automatically taken during the lateral localizer. The whole body scan was performed according to the calculated dose distribution and the initial reference mAs value (mAs_ref_). The scan parameters of the whole body CT were as follows: reference tube current 400 mAs_ref_; tube voltage 120 kVp; rotation time 0.5 s; pitch 0.35; acquisition 128 × 0.6 mm. The actual tube current levels were based on dose modulation with an average adaptation to patient size. After each scan, the effective mAs values (mAs_eff_) according to the effective dose exposure of the whole body scan was documented in an automated dose report.

### Descriptive data

The actual mAs_eff_ values and the CT examination data were extracted from the dose reports for each decedent, which were automatically generated after completing the scan by the CT control software (syngo CT 2012B, release VA44A, Siemens Heathcare, Forchheim, Germany) and automatically sent to and stored in our data archive (syngo.share View, release VA21E, ITH icoserve technology for healthcare GmbH, Innsbruck, Austria). Sex, age (years), actual BW (kg), and estimated time of death were taken from our digital case archive (IBM Notes® 9, release 9.0, Armonk, NY, USA). PMI in days was calculated according to the time period between the estimated time of death and the CT examination date. The presence or absence of metallic medical implants (orthopaedic implants and pacemakers) was noted by reviewing all image data. Actual BW measurements (kg) on the readout of the calibrated floor scale (MultiRange ID5, Mettler-Toledo International Inc., Ohio, Columbus, US) were documented during body intake at our institution, according to our routine protocol.

### Statistical analysis

All statistical analyses were computed using dedicated software (R version 3.3.2., R Core Team, R Foundation for Statistical Computing, Vienna, Austria). A *p* value of < 0.05 was considered to be statistically significant.

## Results

**Fig. 1:**
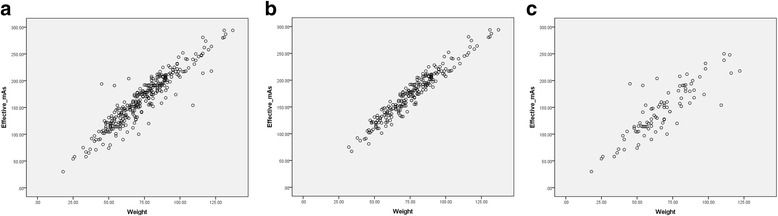
Several outliers are visible for the study population (**a**). However, decedents with a PMI < 4 days (**b**) showed a strong correlation between measured BW and mAs_eff_ values. Of note, all outliers of the study population (**a**) can be assigned to decedents with PMI ≥ 4 days (**c**)

The study population yielded a mean actual BW of 73.8 kg (SD 73.8 ± 20.1 kg, range 18–137 kg) and a mean value of 165.8 mAs_eff_ (SD 165.8 ± 46.4 mAs_eff_, range 30–294 mAs_eff_). The correlation between the measured BW and mAs_eff_ was stronger for PMI < 4 days (*r* = 0.966) than for PMI ≥ 4 days ( *r* = 0.854). The descriptive data and statistical analyses of the study group and of all subgroups are listed in Table 1. The Kolmogorov–Smirnov test showed normal data distributions for all groups and subgroups except females (mAs_eff_, *p* = 0.002; weight, *p* = 0.001) and males (weight, *p* = 0.032) with a PMI < 4 days. The correlation was found to be strong for both females (*r* = 0.974) and males (*r* = 0.960). The same applied to the subgroups for implants (*r* = 0.969) and no implants (*r* = 0.966). All correlation coefficients were statistically significant (*p* < 0.001). Correlations between mAs_eff_ and measured BW for the study population, for PMI < 4 days and for PMI ≥ 4 days are illustrated in Fig. 1.

**Table 1:** Descriptive data and statistical analyses of the study population and of subgroups

	Study population	PMI ≥ 4 days	PMI < 4 days	Women	Men	Implants	No implants
Number of cases	329	89	240	78	162	38	202
Female	105	27	78	78	0	17	61
Male	224	62	162	0	162	21	141
Minimum age	18	22	18	21	18	21	18
Maximum age	95	95	94	94	94	94	94
Mean age	59.0	61.3	58.1	62.0	56.3	69.0	56.1
SD^a^ (±)	18.0	15.8	18.6	18.0	18.7	17.2	18.2
Minimum weight	18	18	32	32	34	34	32
Maximum weight	137	122	137	131	137	120	137
Mean weight	73.8	67.4	76.2	68.4	80.0	75.0	76.4
SD^a^ (±)	20.1	21.0	19.3	20.9	17.2	20.0	19.1
Minimum mAs_eff_	30	30	67	75	67	67	75
Maximum mAs_eff_	294	250	294	294	294	281	294
Mean mAs_eff_	165.8	143.6	174.0	160.5	180.5	174.0	174.0
SD^a^ (±)	46.4	47.3	43.3	48.3	39.0	45.4	42.9
Pearson’s *r* ^b^	0.931	0.854	0.966	0.974	0.960	0.969	0.966
*p* value^c^	<0.001	< 0.001	< 0.001	< 0.001	< 0.001	< 0.001	< 0.001

The study population indicated a strong correlation between measured BW and mAs_eff_ values (*r* = 0.931). The Pearson coefficient was higher for PMI < 4 days (*r* = 0.966) than for PMI ≥ 4 days (*r* = 0.854); *r* was 0.974 for females with PMI < 4 days and 0.960 for males with PMI < 4 days. Further subgroups with PMI < 4 days for implants (*r* = 0.969) and no implants (*r* = 0.966) revealed both strong and nearly equal correlations. All correlation coefficients were statistically significant (*p* < 0.001)

^a^Standard deviation

^b^Pearson correlation coefficient between mAs^eff^ and body weight

^c^ *p* value of the correlation

Multivariate linear regression analysis for PMI < 4 days taking into account the mAs_eff_ (*p* < 0.001), sex (*p* < 0.001) and implants (*p* = 0.271) revealed that sex was a significant factor, whereas implants were not. Therefore, the implants variable was not included in the equation. Based on the results of the multivariate linear regression analysis for PMI < 4 days (constant = 3.732, *p* = 0.007) taking into account mAs_eff_ (B = 0.422, *p* <0.001) and sex (B = −3.108, *p* <0.001), we propose the following linear regression equation to estimate BW:

$$ \mathrm{Estimated}\;\mathrm{BW}=3.732+\left(0.422\times {\mathrm{mAs}}_{\mathrm{eff}}\right)-\left(3.108\times \mathrm{sex}\;\mathrm{index}\right) $$

where the sex index is 0 for males and 1 for females. The SEE was 4.82.

**Fig. 2:**
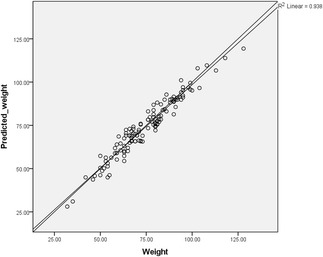
Applying the equation to the validation group revealed a strong correlation between actual BW and predicted BW. The validation group showed no outliers. The coefficient of determination (R^2^) was 0.938

The validation group yielded a mean actual BW of 74.8 kg (SD 74.8 ± 16.7 kg, range 32–128 kg) and a mean mAs_eff_ of 169.5 (SD 168.5 ± 38.3 mAs_eff_, range 65–274 mAs_eff_). The mean predicted BW calculated by the equation was 74.2 kg (SD 74.2 ± 16.6 kg, range 28.1–119.4 kg). Descriptive data of the validation group and of all subgroups are listed in Table 2. The statistical evaluation of data distribution showed normal distributions for the main group and both subgroups. The actual BW and BW predicted by the equation were strongly correlated (*r* = 0.969; women, *r* = 0.972; men, *r* = 0.960). The coefficient of determination (R^2^) was 0.938. The validation group showed no outliers (maximum deviation ±9 kg; mean deviation −0.6 kg; Fig. 2). The Student *t*-test revealed no statistically significant difference between actual BW and predicted BW for the validation group (*p* = 0.119; females, *p* = 0.394; males, *p* = 0.196).

**Table 2:** Descriptive data and statistical analyses for the validation group

	Validation group	Women	Men
Number of cases	125	43	82
Female	43	43	0
Male	82	0	82
Minimum age	18	19	18
Maximum age	96	96	88
Mean age	56.4	59.2	55.0
SD^a^ (±)	18.3	21.7	16.0
Minimum weight	32	32	50
Maximum weight	128	100	128
Mean weight	74.8	66.6	79.1
SD^a^ (±)	16.7	16.9	14.9
Minimum predicted weight	28.1	28.1	50.2
Maximum predicted weight	119.4	101.1	119.4
Mean predicted weight	74.2	66.0	78.5
SD^a^ (±)	16.6	17.6	14.3
Minimum mAs^eff^	65	65	110
Maximum mAs^eff^	274	238	274
Mean mAs^eff^	169.5	154.9	177.2
SD^a^ (±)	38.3	41.8	34.0
Pearson’s *r* ^b^	0.969	0.972	0.960
*p* value (correlation)^c^	< 0.001	< 0.001	< 0.001
*p* value (*t*-test)^d^	0.119	0.394	0.196

Applying the equation on the validation group revealed a strong correlation between measured BW and predicted BW (*r* = 0.969). The Pearson coefficient *r* was 0.972 for females and 0.960 for males. All correlation coefficients were statistically significant (*p* < 0.001). The Student *t*-test revealed no significant difference between actual BW and predicted BW for the validation group (*p* = 0.119; females, *p* = 0.394; males, *p* = 0.196)

^a^ Standard deviation

^b^ Pearson correlation coefficient between actual weight and predicted weight (calculated by the equation)

^c^ *p* value of the correlation

^d^ *p* value of the Student’s *t*-test

## Discussion

This study presents a reliable method to estimate BW using CT dose modulation through a simple equation. We found a strong correlation between BW, measured with the standard scale, and mAs_eff_ values based on CT dose modulation. The proposed equation, taking into account mAs_eff_ and sex, fits 93.8% (R^2^ = 0.938) of the data regarding decedents with PMI < 4 days, without any outliers in the validation group. Thus, a rapid and robust method to determine BW of non-decomposed human decedents is now available. In the forensic setting, this could have value in situations of equipment failure, data loss, or if images were evaluated in isolation. Moreover, this method may have potential in clinical radiology as whole body CT has gained increasing importance in emergency settings such as polytrauma [17–20] or other conditions. Notably, our equation was derived from data obtained with the CT scanner and the protocol we used and may not provide the same results when different CT models from other vendors and other protocols are used. However, this study clearly describes how institutes can calculate an equation for their own whole body CT unit and protocol.

The study population was divided into cases with PMI < 4 days and cases with PMI ≥ 4 days, because of decomposition- or putrefaction-related changes. This temporal separation was chosen based on the experiences of our forensic pathologists. Although decomposition is dependent on several factors [21], in our temperate climate, generalized bloating usually starts to appear after 72 h after demise [16]. For this study the chosen point of time for temporal separation seemed appropriate. All decedents with PMI < 4 days showed an excellent correlation between mAs_eff_ and BW (*r* = 0.966). By contrast, the correlation was weaker in decedents with PMI ≥ 4 days (*r* = 0.854), probably due to decomposition- or putrefaction-related changes (e.g. gaseous distention or putrefaction fluid). It is conceivable that decedents with a shorter PMI (or living patients) may show even higher correlation between mAs_eff_ and BW. In the field of postmortem imaging, Jackowski et al. [9] presented a method using postmortem CT. The method was derived from a study by Abe et al. [22], who calculated a soft tissue multiplication factor for detecting whole body skeletal muscle mass in the living. Based on 50 cases (30 adults and 20 paediatrics) with a short PMI (not described more accurately), Jackowski et al. [9] calculated a multiplication factor to estimate BW of decedents based on whole body segmentation. However, whole body segmentation requires specialized skills and software and additional imaging processing steps and can be time consuming. Conversely, the use of dose-modulated mAs and an equation enable rapid BW estimation.

Rapid BW calculation based on dose modulation for adult patients may show potential in emergency radiology with respect to drug dosage or dosage of contrast media, which are usually based on patient BW. Fernandes et al. [1] demonstrated that 33% of estimates from physicians and nurses deviate by more than 10% from actual BW of ambulatory patients (indicated with a 95% confidence interval). As mentioned by the authors, BW estimates for patients in the supine position may be even less accurate. An equation by Buckley et al. [2] yielded greater accuracy compared to visual BW estimates made by physicians and nurses. Deviations greater than ±10 kg from measured BW still occurred in 15% of male patients and 27% of female patients. Thus, the authors recommended the linear regression equation only for male patients when patients are not able to state their BW. By contrast, the present study revealed strong correlations for both females and males with a PMI < 4 days. However, the data of each of these two subgroups were not normal distributed; therefore, the results are less robust. The mean BW of males (80.0 kg) was in the range of the standard reference patient BW of 70–80 kg used in dose modulation software and revealed a strong correlation (*r* = 0.960). Despite the fact that the mean BW of females (68.4 kg) was below the range of the standard reference patient BW of 70–80 kg, the correlation was also strong (*r* = 0.974). Although, metallic implants affect x-ray attenuation [23], the correlation between decedents with implants (*r* = 0.969) was nearly equal to decedents without implants (*r* = 0.966). In contrast to sex, taking implants into account was not statistically significant in the multivariate linear regression analysis; thus, implants were not considered as a factor to consider. Therefore, the presence or absence of metallic implants was not taken into account in the linear regression equation. We hypothesize that small medical devices may also have little influence on the correlation.

In our study, the applied dose modulation (CARE Dose 4D™) was used with an average adaptation to patient BW. CARE Dose 4D™ also allows for different adaptation options regarding patient size (very strong, strong, weak, and very weak), which can be selected for adult slim or adult obese patients. Different adaptation settings result in different mAs_eff_ values [15]. Therefore, changes in adaptation options would result in different correlations between mAs_eff_ and patient BW. We hypothesize that separate equations for slim or obese patients using weak or strong adaptations, respectively, will result in more precise BW estimations. Further, dose modulation was based on a lateral whole body localizer. Our postmortem CT protocol included at first a frontal localizer and afterwards a lateral localizer. The correlation between dose modulations based on attenuation measurements during the frontal localizer and BW was not evaluated in this study. However, this study clearly describes the calculation of an equation for BW estimation based on mAs_eff_ values, which can be easily calculated for any clinical CT protocol using dose modulation.

Admittedly, this study has several limitations when considering the clinical perspective. First, our results are based on a standardized postmortem CT protocol according to the literature [12]. Radiation dose to the decedent can be neglected in postmortem imaging; therefore, a high mAs_ref_ value of 400 is standard for whole body scans. Further studies are required regarding mAs_eff_ values from clinical protocols. Second, the estimation of BW based on CT using dose modulation requires a whole body scan. Therefore, this approach is limited to polytrauma patients who undergo whole body CT scans. Third, automatic exposure control systems are available from several CT vendors [13, 14] but dose modulation strategies vary between vendors. The results of this study are based on the dose modulation strategy of a single vendor. However, we hypothesize that other vendors provide similar correlations, which can be investigated in the same way as the present study.

To summarize, this study demonstrates a rapid and reliable method for BW estimation. Given the lack of reliable methods for practitioners to estimate patient BW based on visual parameters or physical exam, BW estimation based on CT dose modulation may have potential use in clinical radiology and polytrauma patients. Certainly, further studies are required.
